# *Don’t stand so close to me:* Microbiota‐facilitated enemy release dynamics in introduced *Onthophagus taurus* dung beetles

**DOI:** 10.1002/ece3.6836

**Published:** 2020-11-19

**Authors:** Erik S. Parker, Armin P. Moczek

**Affiliations:** ^1^ Department of Biology Indiana University Bloomington IN USA

**Keywords:** introduced species, microbiome, range expansion

## Abstract

Microbial symbionts can influence their hosts in stunningly diverse ways. Emerging research suggests that an underappreciated facet of these relationships is the influence microbes can have on their host's responses to novel, or stressful, environmental conditions. We sought to address these and related questions in populations resulting from the recent introduction and subsequent rapid range expansion of *Onthophagus taurus* dung beetles. Specifically, we manipulated both microbial communities and rearing temperature to detect signatures of developmental and life history differentiation in response to the local thermal conditions in two populations derived from the southern most (Florida) and northern most (Michigan) extremes of the exotic Eastern U.S. range of *O. taurus*. We then sought to determine the contributions, if any, of host‐associated microbiota to this differentiation. We found that when reared under common garden conditions individuals from Florida and Michigan populations differed significantly in developmental performance measures and life history traits, consistent with population divergence. At the same time, and contrary to our predictions, we failed to find support for the hypothesis that animals perform better if reared at temperatures that match their location of origin and that performance differences may be mediated by host‐associated microbiota. Instead, we found that microbiome swapping across host populations improved developmental performance in both populations, consistent with enemy release dynamics. We discuss the implications of our results for our understanding of the rapid spread of exotic *O. taurus* through the Eastern United States and the significance of symbiosis in host responses to novel environmental conditions more broadly.

## Graphical abstract

Microbial symbionts can influence their hosts in stunningly diverse ways, and we were interested in how these symbionts can influence their host's responses to novel, or stressful, environmental conditions. We sought to address this question by manipulating both microbial communities and rearing temperature in two populations of recently invasive Onthophagus taurus dung beetles. We failed to find support for our hypothesis that animals perform better if reared at temperatures that match their location of origin and that performance differences may be mediated by host‐associated microbiota—instead, we found that microbiome swapping across host populations improved developmental performance in both populations, consistent with enemy‐release dynamics.

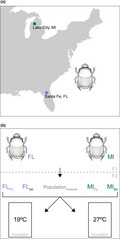

## 1 INTRODUCTION

Responding to changing environmental conditions requires organisms to either plastically shift patterns of phenotypic expression within a lifetime or undergo adaptive evolution across multiple generations (Barrett & Schluter, 2008; West‐Eberhard, 2003). The individual contributions of these two mechanisms and their potential synergistic effects are of particular interest as we consider the impacts of anthropogenic climate change, especially as they relate to crucial ecosystem service providers (Kingsolver & Buckley, 2017; Merilä & Hendry, 2014; Mooney et al., 2009). However, what is less well understood is how microbial symbionts might ameliorate both plastic and adaptive responses of their hosts when confronted with novel, or stressful, environmental conditions. Such microbiome‐mediated ecological adaptation has recently been hypothesized to be a relatively common occurrence (Sudakaran et al., 2017), and experimental evidence across a number of taxa has begun to support that the formation of novel, or evolution of existing, host–symbiont relationships may facilitate rapid host adaptation and range expansion (e.g., *Sirex* woodwasps: Wooding et al., 2013; Hajek et al., 2013; ants: Mueller et al., 2011; Cheng et al., 2019; mice: Chevalier et al., 2015; pine trees: Gundale et al., 2016; and *Brassica* plants: Lau and Lennon, 2012). Despite these advances, however, assessing causality in the patterns uncovered remains challenging.

In order to more directly investigate the extent to which microbial symbionts themselves facilitate host plasticity and adaptation in the face of environmental change, additional studies are needed where both the microbiome and environmental conditions of a host are directly manipulable. We sought to address this challenge in the bull‐headed dung beetle *Onthophagus taurus*, which was introduced from its native Mediterranean range into both Eastern and Western Australia, as well as the Eastern United States (Silva et al., 2016). Introductions into Australia were part of a biocontrol effort to combat dung breeding flies and pasture degradation starting in the late 1960s, required beetles to be surface sterilized as eggs and quarantined for a generation prior to release, followed by extensive reharvesting and redistributing in the field to increase the species’ introduced range (Edwards, 2007). In contrast, in the Eastern United States *O. taurus* appears to have been introduced accidentally from an unknown source population (Vulinec & Eudy, 1993). Since the first documentation of this species in Santa Rosa County, Florida, in 1971, and without the subsequent aid of deliberate redistribution efforts, *O. taurus* managed to spread to Texas in the west, and the Canadian border in the north, ultimately occupying a climatic niche space far exceeding that of both its native Mediterranean and introduced Australian counterparts (Floate et al., 2017; Rounds & Floate, 2012; Silva et al., 2016). However, exactly how EUS *O. taurus* populations were able to disproportionately expand their climatic niche is unclear. Here, we test the hypothesis that the expansion of *O. taurus* in the Eastern United States was facilitated through local adaptation of beneficial host–microbiome interactions.

Entomologists have long hypothesized that *Onthophagus* beetles are able to feed on their characteristic diet of nutritionally challenging ruminant dung through associations with symbiotic microbes (Frank et al., 2017; Goidanich & Malan, 1962; Holter, 2016; Rougon et al., 1990). Recent research increasingly supports this hypothesis. Onthophagine beetles reproduce via the construction of subterranean brood balls, compact, spherical constructions of dung with an egg chamber containing a single egg within. Work in *O. taurus* has shown that the gut microbial communities of mothers and their larval offspring are highly similar, and that this similarity arises because mothers directly pass their gut microbes to their offspring through a fecal secretion—called the “pedestal”—positioned underneath the egg and consumed by larvae immediately after hatching (Estes et al., 2013). Subsequent work also showed that (a) vertically transmitted pedestal microbes are developmentally important, as *Onthophagus* beetles reared without their pedestals take longer to develop and eclose to smaller adults as compared to conspecifics provided their pedestals during the larval stage (Schwab et al., 2016); (b) these negative growth consequences are exacerbated under stressful environmental conditions but may be rescued through inoculation with pedestal‐derived bacterial cultures (Schwab et al., 2016); and (c) the microbial communities of *Onthophagus* beetles are diverse and structured both by ancestral associations and environmental forces which have brought about shifts in microbiome composition in as short as 50 years following the introduction of *O. taurus* into the Eastern United States and Australia (Parker et al., 2020). We thus hypothesized that the successful range expansion seen specifically in Eastern United States *O. taurus* may be due at least in part to local adaptations in the relationship between beetle hosts and their associated microbiota.

To address this hypothesis, we explored the importance of the pedestal microbiota on developmental outcomes of fitness‐related traits including development time, survival rate, and adult body size in two populations of EUS *O. taurus* beetles from Northern Florida (FL) and Northern Michigan (MI)—the southern and northern extremes of the species’ current EUS range. Specifically, we assessed the following: (a) whether beetles derived from these two populations exhibit divergence in the thermal sensitivity of their development, (b) whether both populations show signatures of local adaptation to thermal conditions by rearing both FL and MI animals at both FL and MI‐like soil temperatures; and (c) whether pedestal‐derived microbiota facilitate local thermal adaptations by enhancing host fitness in challenging temperature conditions.

## 2 MATERIALS AND METHODS

### 2.1 Beetle collection and husbandry

**FIGURE 1:**
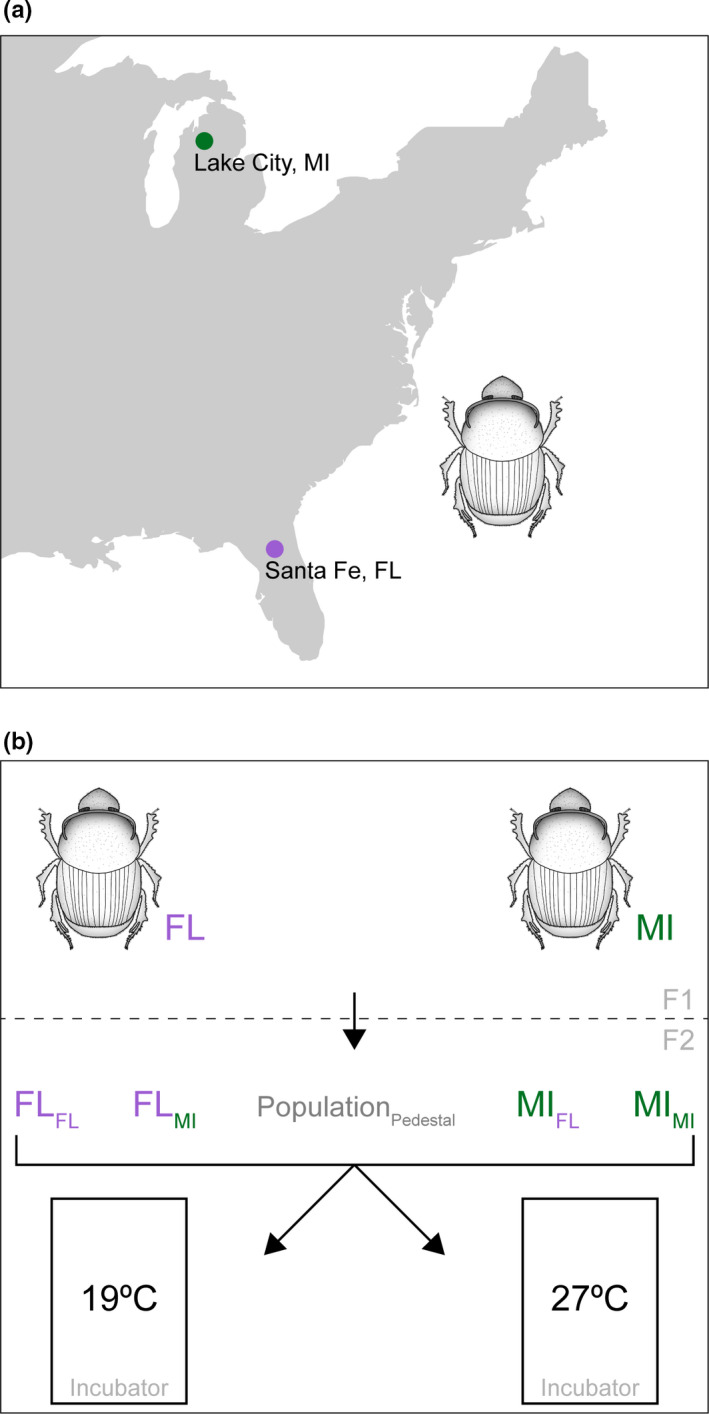
Collection sites and experimental design. (a) Field collection sites used for this study. Santa Fe, FL and Lake City, and MI mark the southern and northern extremes of the *Onthophagus taurus* distribution in the Eastern United States, respectively. (b) Diagram of experimental procedure. F1 animals were used to generate eggs for experimental manipulation. F2 eggs were assigned to either their own pedestal (self‐) or pedestals derived from the other population (cross‐inoculated). Animals from all four experimental groups were then reared at either 19ºC (MI conditions) or 27ºC (FL conditions)

*Onthophagus taurus* beetles were field collected from two locations in the Eastern United States representing their current southern and northern extremes of their range, and then shipped to Bloomington, IN. In the south, beetles were collected from the UF Santa Fe River Ranch Beef Unit, near Alachua, Florida (29.9242, −82.4950) in early May 2019; and in the north, beetles were collected from the MSU Lake City Research Center, Lake City, Michigan (44.3089, −85.2034) in late August 2019 (Figure 1). After arriving in the laboratory, all beetles were transferred into single‐population colonies, where they were maintained in a sand/soil mixture at 24°C and fed antibiotic‐free cow dung twice weekly as described in Moczek (2006). Because of differences in collection times between the two populations, animals were reared for one generation in the laboratory before they were used for experiments.

To breed animals for experiments, seven adult females and three adult males were allowed to mate and produce brood balls in plastic containers (25 cm × 25 cm × 13 cm) filled with a moist sand/soil mixture and provided dung ad libitum. Following protocols described in Parker et al. (2018), brood balls were collected after six days, carefully opened with gloved hands, and eggs inside extracted using autoclave sterilized paintbrushes. Eggs were then surface sterilized with one rinse of 100 µl of 1% bleach and 0.1% Triton X‐100 solution, followed by two rinses of 1 ml of deionized water. Following this, the maternal fecal deposit to which the egg was oviposited (the aforementioned pedestal) was dissected out of the brood ball using a flame‐sterilized surgical blade. This pedestal was then placed into the center of an artificial brood ball constructed within the well of a twelve‐well plate, and a single sterile egg was placed on top all following Parker et al. (2018). Eggs obtained from each population were haphazardly assigned to one of two treatments within each plate: a self‐inoculated treatment where each sterilized egg was placed back on its own pedestal, or a cross‐inoculated treatment where eggs were placed on a pedestal from the other population. These four resulting treatment groups were blocked vertically within each plate, and their order was randomized to minimize within‐plate effects, with three individuals per treatment group in each plate.

Furthermore, each plate was haphazardly assigned to one of two temperature treatments. Plates were stored in an incubator, at either 19 or 27°C for all of development. These temperatures were chosen to mimic peak breeding season soil temperatures at the MI and FL collection locations, respectively, as obtained from long‐term monitoring records (from Syngenta, the National Oceanic and Atmospheric Administration, and the US Department of Agriculture National Resources Conservation Service). Plates were then checked once every 48 hr to assess animal growth and stage of development. After each check, the orientation and position of plates within the incubators were changed to further minimize the effects of any potential microclimatic variation within the incubator. Final sample sizes were 30 individuals per treatment at 19°C and 27 per treatment at 27°C.

### 2.2 Data collection

To assess the effects of pedestal swapping, and our temperature treatments on the growth, development, and survival of our animals, we collected the following measurements for each individual: days until (a) final (third) larval instar, (b) pupation, and (c) adulthood. We also measured the weight of our animals at two timepoints during their development: We first measured larval mass 7 days after an individual was first scored as a third instar. By this time, larvae are nearing the peak weight they will obtain during their larval growth period, and we use this measure as an indication of a given larva's ability to maximize mass gain during a 7‐day period. We also assessed pupal mass 48 hr after an individual was scored as a pupa as an estimate of final body mass acquisition after larvae have purged their gut and successfully completed the larval to pupal molt. Pupal mass is typically very closely correlated with adult body size (Moczek, 2006). All mass measurements were recorded to the nearest 0.0001 g using a Mettler Toledo AL54 (Mettler, Columbus, Ohio, USA) scientific scale. All animals who reached the pupal stage were sexed to allow for analysis of sex differences in treatment effects. Finally, we also measured time to death for animals that did not survive to adulthood, survival rates, and adult body size (as pronotum width, using a digital camera and ImageJ software as previously described (Moczek, 2006) whenever applicable).

### 2.3 Data analysis

All analyses were performed in R v3.5.3 (R Core Team, 2013) and RStudio (RStudio Team, 2015) using the packages *car* (Fox et al., 2012), *GGally* (Schloerke et al., 2017), *ggplot2* (Wickham, 2016), and *visreg* (Breheny & Burchett, 2017).

To investigate the specific influence of our pedestal manipulations on the various growth, development, and survival metrics measured, we constructed linear mixed and generalized linear mixed (binomial family error distribution) models regressing these measured variables on all possible main effect combinations, and interactions of pedestal treatment, population, rearing temperature, and sex. Plate code was included as the random effect in each model to account for random error introduced by our experimental design. The regressors in each model constructed were validated using Wald chi‐square tests, and regression diagnostics were performed to check assumptions related to normality of the residuals, homoscedasticity of the variance, and for the presence of outliers or otherwise overly influential points. Nonsignificant interaction terms were removed, and all higher‐order interactions above two‐way were never significant.

Furthermore, Levene's tests were used to check for equality of variances between measured variables for our different sample groups. The Kaplan–Meier estimator was used to obtain survival curves for each of our eight treatment groups, and the log‐rank test was used to compare these curves.

## 3 RESULTS

In this study, we sought to investigate potential differences in growth, development, and survival between *Onthophagus taurus* beetles across the extremes of their Eastern US range—and to examine to what extent these differences can be attributed to the pedestal microbiome (the primary source of vertical microbial transmission in this genus; Estes et al., 2013; Schwab et al., 2016). To do so, we employed a fully factorial experimental design where we manipulated both the rearing temperature (19 or 27°C reflecting peak breeding season soil temperatures at each location) and pedestal origin (self‐ or cross‐inoculated) of beetles from both Northern Michigan (MI) and North‐Central Florida (FL). Our predictions for this experiment were multilayered. First, we expected significant differences in developmental performance metrics between MI and FL populations when reared with their own pedestal (self‐inoculated) depending on rearing temperature. Specifically, we expected MI individuals to outperform FL individuals at 19ºC, but the inverse to manifest at 27°C. Second, we predicted that our pedestal manipulation would interact with rearing temperature and population to increase fitness in a subset of situations. We found partial support for these predictions.

### 3.1 Population origin affects developmental performance and survival, irrespective of rearing temperature

**FIGURE 2:**
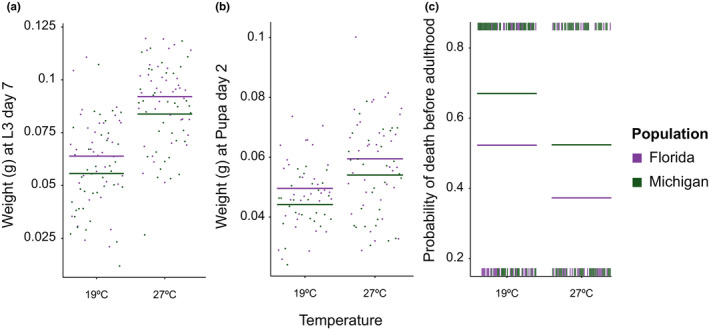
Effect of population of origin and rearing temperature on development and survival. Effect plots showing the estimated influence of population of origin, and rearing temperature on (a) weight at day 7 of the final larval instar (*n = *151), (b) weight at day 2 of the pupal stage (*n* = 125) and (c) probability of death before adulthood for all animals (*n* = 228). All plots were derived from either linear mixed (a and b) or generalized linear mixed (c) models containing the factors rearing temperature, population of origin, pedestal treatment, and random factor of plate code. Animals from the FL population (regardless of temperature) and animals reared at higher temperatures (regardless of population) showed higher fitness for both measured variables. Points (a and b) indicate partial residuals, vertical dashes (rug plots in c) indicate individual datapoints in each group, and horizontal colored lines indicate predicted value in each plot

FL and MI populations differed significantly in several developmental performance measures and life history traits, consistent with population divergence. At the same time, and contrary to our predictions, we failed to find support for our hypothesis that animals perform better if reared at population‐specific rearing temperatures and that performance differences may be mediated by pedestal‐derived microbiota. Specifically, we found that FL larvae and pupae grew to larger sizes and survived at a higher rate compared to MI larvae (Figure 2; Table 1). These effects were seen in linear mixed and generalized linear mixed models which considered rearing temperature and pedestal treatment in addition to population of origin. In addition to the significant difference seen between MI and FL animals, we observed increased larval and pupal mass, as well as larger adult body sizes and increased survival rates for both populations when reared at 27°C (Figure 2; Table 1). In contrast, we saw no significant difference in either larval mass or survival rate between cross‐ and self‐inoculated animals (Table 1).

**TABLE 1:** Mixed models testing for the significance of rearing temperature, population of origin, and pedestal treatment, on fitness‐related developmental metrics. 12‐well plate code was used as a random effect in each model. Nonsignificant interactions were removed, and three‐way interactions were never significant. Columns show chi‐squared test statistic values, resulting test probabilities, and estimated effect sizes and standard error for each regression term

	Larval weight			Pupal weight			Adult size			Survival			Total development time		
	*Χ^2^*	*p*	*β* ± *SE* (g)	*Χ^2^*	*p*	*β* ± *SE* (g)	*Χ^2^*	*p*	*β* ± *SE* (mm)	*Χ^2^*	*p*	*β* ± *SE* (prob.)	*Χ^2^*	*p*	*β* ± *SE* (days)
Population	5.86	**.015**	−0.0082 ± 0.0034	5.643	**.018**	−0.0054 ± 0.0025	1.101	.294	−0.076 ± 0.073	5.135	**.023**	0.65 ± 0.27	1.067	.302	−0.84 ± 0.81
Pedestal	1.22	.269	−0.0038 ± 0.0034	0.256	.614	−0.00037 ± 0.0029	0.076	.783	0.02 ± 0.072	0.166	.684	0.47 ± 0.27	3.039	.081	3.23 ± 1.19
Temperature	28.769	**<.001**	0.028 ± 0.0053	11.698	**<.001**	0.0099 ± 0.0033	14.512	**<.001**	0.29 ± 0.076	4.952	**.026**	0.35 ± 0.28	412.219	**<.001**	−20.88 ± 1.37
Temperature X pedestal													4.36	**.037**	−3.35 ± 1.61

Despite these differences early on during development, we failed to detect a significant influence of population origin on final adult body size (Table 1). Likewise, even though we saw a significant difference in ultimate survival rate between these two populations there was no significant difference in the slope or shape of their survival curves—as given by the Kaplan–Meier estimator and corresponding log‐rank test.

### 3.2 Microbiome swapping across host populations improves developmental performance in both populations, but only at one rearing temperature

**FIGURE 3:**
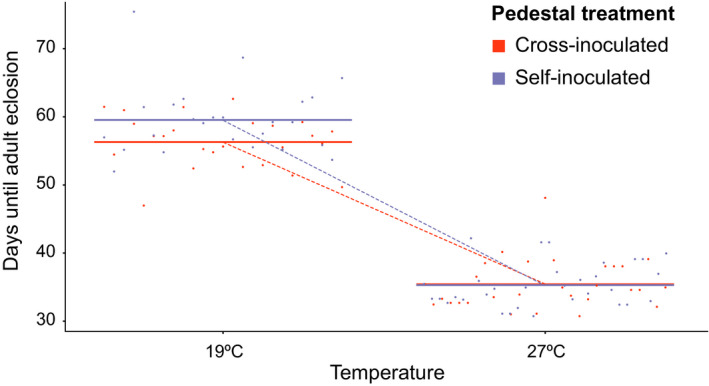
Effects of pedestal manipulation. Effect plot showing the estimated influence of pedestal manipulation on days until adult eclosion (*n* = 111). Generated from a linear mixed model containing the factors rearing temperature, pedestal treatment, population of origin, the random factor plate code, and the interaction between pedestal treatment and rearing temperature. Animals which received the other population's pedestal (cross‐inoculated) reached adulthood faster than animals which received their own pedestal (self‐inoculated), but only at 19ºC. Points indicate partial residuals, and colored lines indicate predicted value. Diagonal, dotted lines added to help denote the significant interaction between temperature and pedestal treatment as visualized by the difference in slopes between treatment groups

We originally predicted that animals from either population would perform better when reared with their own pedestal microbes. However, we observed precisely the opposite pattern, though only at one of the two rearing temperatures. Larvae derived from both FL and MI populations who received their own pedestal (self‐inoculated) developed significantly slower than cross‐inoculated larvae (~3 days) at 19°C, but not at 27°C (Figure 3; Table 1 **)**. However, as previously noted we saw no significant difference caused by pedestal manipulation in the size of these animals at any life stage (Table 1), that is, in a linear mixed model explaining total development time (egg to adult eclosion) by pedestal treatment, animal population, rearing temperature, and the interaction between rearing temperature and pedestal treatment, the cross‐inoculation treatment significantly reduced the time needed to reach adulthood at 19°C only, but did not affect the size of animals at either of these life stages. Importantly, population of origin did not affect this pattern as both MI and FL beetles developed faster when subject to the cross‐inoculation treatment (Table 1). Furthermore, the interaction between population and pedestal treatment was not significant, meaning cross‐inoculation reduced total development time to the same degree in both MI and FL populations at 19°C. Lastly, we detected no significant differences between male and female individuals for any of the metrics we measured.

## 4 DISCUSSION

In this study, we leveraged the rapid range expansion of the bull‐headed dung beetle *Onthophagus taurus* in the Eastern United States to address whether host‐associated microbiota can mediate local thermal adaptation and host range expansion. We sought to address this question using an experimental design which manipulated both the microbial and developmental thermal environment of larvae derived from two populations representing the southern and northern extremes of the latitudinal range this species have recently established in the Eastern United States (Figure 1). Below we discuss the most important implications of our results.

### 4.1 Florida‐derived beetles outperform Michigan‐derived beetles regardless of rearing temperature

Based on earlier studies documenting rapid population differentiation in *O. taurus* (Beckers et al., 2015; Casasa & Moczek, 2018; Moczek, 2003), and the large climatic differences experienced by these beetles over their Eastern US range (Silva et al., 2016), we predicted that populations collected at the southern and northern extremes of this range would show significant divergences in developmental performance and/or life history. We found that in support of these predictions, populations from FL and MI diverged both in adult survival rate and larval size (Figure 2). At the same time, we were unable to find support for our second prediction that populations would show local adaptation to their respective local thermal conditions as FL‐derived beetles outperformed MI‐derived beetles regardless of rearing temperature (Figure 2). This is in contrast to a recent study documenting clinal differentiation and the evolution of genotype‐by‐environment interactions across Eastern US *O. taurus* populations (Rohner & Moczek, 2020), which, however, assessed four populations, was able to use the offspring of field collected individuals which possibly introduced direct maternal effects that could not be accounted for, and did not require the experimental manipulation of pedestals. Together, these factors might explain the disagreement in findings between these two studies.

### 4.2 Exchanging pedestal microbiota between populations speeds growth at one rearing temperature, consistent with enemy release dynamics

In line with our general predictions, we found that pedestal–microbiome manipulation significantly impacted fitness‐related traits in a subset of environmental conditions and genetic backgrounds. However, our specific prediction that this impact would be fitness enhancing under thermal conditions reflective of the source population was not met. Instead, we found that providing both MI and FL animals with the other population's pedestal shortened larval development time (yet without affecting final adult body size; Figure 3; Table 1), a trait directly linked to reduced generation time and increased fitness in many insects (Kingsolver & Huey, 2008). This finding was unexpected because previous research demonstrated that both (a) withholding pedestals (Schwab et al., 2016) and (b) pedestal swaps across species (Parker et al., 2018) result in negative developmental outcomes and (c) that *O. taurus* populations obtained from different exotic ranges—while maintaining a putative core microbiome—also harbor taxonomically distinct microbial communities (Parker et al., 2020). Collectively, this raises the possibility that host–microbiota co‐adaptation may not manifest on the level of populations *within* a given range. Instead, our finding that cross‐inoculated individuals outperform self‐inoculated individuals raises the alternative hypothesis that this enhanced performance occurred because host individuals may have been released from pressures imposed by microbial pathogens while still maintaining a functional core microbiome.

The enemy release hypothesis posits that one reason why non‐native species often outperform their native counterparts is that they have been released from the pressures imposed by natural enemies (such as parasites, predators, or microbial pathogens) in their native range (Mitchell et al., 2006; Reinhart & Callaway, 2006). While most commonly invoked in plant systems, this hypothesis is equally applicable to animal systems—and in fact patterns consistent with this hypothesis have been observed in a number of animal taxa (Marr et al., 2008; Ross et al., 2010; Torchin et al., 2003). Furthermore, growing evidence highlights the context‐dependent nature of host–microbe relationships. Microbial symbionts can evolve mutualistic relationships with their hosts under certain contexts, but as those conditions change—that is, if a pathogen does not occur in a newly colonized host environment, or if a host's diet changes—these relationships can shift and become neutral or even deleterious to host fitness (Corbin et al., 2017; Gerardo & Parker, 2014). Our results are consistent with a scenario whereby pedestal microbiota exchange between MI and FL *O. taurus* populations resulted in a release from negative pressures which in turn lead to accelerated host development (Kingsolver & Huey, 2008). If correct, these findings raise the possibility that host range expansions as seen in *O. taurus* may be facilitated not only by the acquisition of beneficial microbial interactions, but also by the location‐specific removal of *negative* microbial challenges. Future studies comparing pathogen loads of various *O. taurus* populations from both their native Mediterranean and exotic Eastern US ranges would help to directly test this hypothesis.

Finally, it is worth noting that microbiome swapping enhanced larval development of both populations, yet at only one temperature, the Michigan like 19°C, but not the 27°C meant to reflect Florida soil temperatures. This suggests that the interactions between host and microbial physiology that influence development time and growth, whatever those may be, are themselves temperature sensitive. This may not be that surprising, however, because on one side a robust body of work has already demonstrated the temperature dependence of fitness relevant traits in *Onthophagus* (e.g., development time, size at pupation, and eclosion success; Floate et al., 2014; Macagno et al., 2016; Macagno et al., 2018; Rohner, Macagno, & Moczek, 2020), while on the other diverse aspects of the external environment, including temperature, are well known to impact host–microbiome interactions in other systems (Renoz et al., 2019). Combined, our results thus raise the possibility that the relatively slow host metabolism and growth possible at 19°C may allow population‐specific microbiome members to exert their growth limiting effects, whereas the more rapid host metabolism and growth possible at 27°C may override the influences of individual microbiome members regardless of their specific origin, hypotheses that clearly warrant further scrutiny.

## 5 CONCLUSION

Understanding how animals respond to environmental conditions is of the utmost importance in a rapidly changing world. The role and significance of host‐associated microbiota in this context remain understudied (Sudakaran et al., 2017). Our results provide an example of the complex ways in which changes in host–microbiota associations may limit or facilitate successful range expansions. Specifically, our work raises the possibility that successful range expansions in dung beetles, rather than being facilitated through the acquisition of beneficial microbial interactions may in addition, or instead, be enabled by the release from *negative* microbial challenges. Though more work is clearly needed to assess this particular hypothesis, our results underscore how host–microbiome interactions may complicate host responses to environmental change.

## CONFLICT OF INTEREST

The authors declare no conflicts of interest for this work.

## AUTHOR CONTRIBUTION

**Erik S. Parker:** Conceptualization (equal); Data curation (equal); Formal analysis (lead); Investigation (lead); Methodology (equal); Project administration (lead); Writing‐original draft (lead); Writing‐review & editing (equal). **Armin P. Moczek:** Conceptualization (equal); Funding acquisition (lead); Resources (lead); Supervision (lead); Validation (lead); Writing‐review & editing (equal).

## Data Availability

Data available on Dryad under https://doi.org/10.5061/dryad.69p8cz903.
